# Annotations

**Published:** 1906-06-02

**Authors:** 


### ANNOTATIONS.

### A Practical Lesson of Cancer Research.

Although up to the present the impetus recently given to cancer-research has not, unhappily, provided any royal road to the cure of the malady, the Imperial Cancer Research Fund has done much useful work in dispelling false traditions and faulty hypotheses. Such negative results do not command enthusiasm, but it is obvious that any scientific investigation of a baffling problem must include a painstaking consideration of such hypotheses as carry any show of reason, in order that they may be once and for all dismissed if they prove inadequate This is a thankless task at best, and, since it is a necessary one, meritorious to a corresponding degree. This being so it is pleasant to be able to welcome a practical outcome of the research, which though again negative, affords a clinical lesson of great value. It consists in the experimental demonstration that malignant new-growths, *per se*, have no symptomatology whatever. By dint of the artificial propagation in mice of Jensen's tumour, an adeno-carcinoma, Dr. Bashford has made it quite certain that the cachexia associated with malignant disease depends upon one of two accidents, and is not inherently a phenomenon of the growth of cancer. These accidents are either some mechanical interference with a vital process, such for instance as occlusion of the intestine, or rupture of the integument of the growth and resulting ulceration. The clinical bearing of this fact becomes clear when we consider that at present there is no known cure for cancerous growths except complete extirpation with the knife. If nothing but microscopy can reveal the malignancy of cancerous growths in their early stages, no time should be lost in submitting to microscopic examination the tissues of any suspicious tumour. The possible superfluousness of a small operation is a trifle compared with the neglect of a golden opportunity which can never recur.

### Premature Burial.

Under the heading of "Startling Figures Supplied by a Doctor," one of the daily papers comments upon a meeting of the London Society for the Prevention of Premature Burial. The doctor referred to is the chief spokesman of another society, one of those societies which purport to act on behalf of animals, but might more correctly be termed societies for the spread of misguided sensationalism. "Startling figures" they indeed present for public consumption, but "startling" mainly on account of their careless disregard for truth. Some men endowed with a glib tongue delight to win a high place in the favour of those who possess ears "itching" to hear something which will stir their emotions, and no doubt the speaker on premature burial, who has spent so much of his life in dealing with the sensational, found the subject well suited to his gifts. Although we have no sympathy with an exaggerated presentation of facts, it is interesting to note that apparently premature burial is not entirely a myth. In the words of a distinguished English physician "the most ghastly incidents of fiction have been paralleled by well-authenticated facts." Accounts which can scarcely be called ghastly, but are certainly remarkable, are those given of the deathlike trance into which Indian fakirs are said to voluntarily enter. While, however, it appears that a condition may occur in which so far as external signs are concerned death is closely simulated, such a condition is extremely rare. It may be added that this simulated death is akin to hysteria, and no doubt can be produced by suggestion. Probably one of the ways most calculated to produce an example would be to work upon the fears of nervous people by presenting to their imaginations delineations of real or fictitious occurrences of deathlike trance. If instances occur in the near future in England they will probably be amongst the members of the Society for the Prevention of Premature Burial.

### The Worth of Books.

In a recent article in one of our contemporaries the writer indulges in an editorial lament over the "weakness" of the "present-day supply of medical books and journals," and, with a pathetic and ingenuous confidence in the power of authority, calls for the establishment of a central clearing house through which "only works of real value" would be allowed to pass into circulation. Neither the matter nor the manner of modern publications escapes our critic's censure, though his tears over grammatical errors would perhaps be more effective were his own composition beyond reproach. Fortunately there is not the slightest chance that his proposals will be realised, and therefore we may still hope to be able to make our own choice of books instead of depending upon the wisdom and discretion of a committee of superior persons. For our contemporary, it seems, this will mean "that pleasure becomes a toil and study a work of disentanglement"; but serious as these consequences may be for him, we do not profess much sympathy. What seems to be forgotten is that an arrangement which may be necessary for schoolboys and undergaduates would be a gratuitous impertinence when applied to grown men. We by no means say that free and unlimited writing and publication have no disadvantages, but we do say that these are more than counterbalanced by the attendant benefits. There is no doubt that the worth of a book or article, at least to any person of discrimination and judgment, may be considerable even though the matter generally is poor and the composition contemptible. A new idea may be found even in a bad book, and whilst we would deal faithfully with the author when the opportunity occurred, we cannot sympathise with the proposal that he should be suffocated in a clearing house. Let him have his say, and we are confident he will ere long find his level. All this talk about restricting publication and about modern decadence is as old as the hills, and merely indicates forgetfulness of the fact that books are of different classes and exist for different purposes—"some are to be tasted, others to be swallowed, and some few to be chewed and digested."

